# Identification of Novel Conotoxin Precursors from the Cone Snail *Conus spurius* by High-Throughput RNA Sequencing

**DOI:** 10.3390/md19100547

**Published:** 2021-09-28

**Authors:** Roberto Zamora-Bustillos, Mario Alberto Martínez-Núñez, Manuel B. Aguilar, Reyna Cristina Collí-Dula, Diego Alfredo Brito-Domínguez

**Affiliations:** 1 División de Estudios de Posgrado e Investigación, TecNM/Instituto Tecnológico de Conkal, Av. Tecnológico S/N, Conkal 97345, Yucatán, Mexico; L17830114@china.tecnm.mx; 2 Unidad Multidisciplinaria de Docencia e Investigación, UMDI-SISAL, Facultad de Ciencias, Universidad Nacional Autónoma de México, Sierra Papacal-Chuburna Km 5, Mérida 97302, Yucatán, Mexico; mamn@ciencias.unam.mx; 3 Laboratorio de Neurofarmacología Marina, Departamento de Neurobiología Celular y Molecular, Instituto de Neurobiología, Universidad Nacional Autónoma de México, Juriquilla 76230, Querétaro, Mexico; maguilar@unam.mx; 4 Departamento de Recursos del Mar, Cinvestav Unidad Mérida, Mérida 97310, Yucatán, Mexico; rcolli.dula@cinvestav.mx

**Keywords:** *Conus spurius*, conopeptide precursors, superfamily, transcriptome

## Abstract

Marine gastropods of the genus *Conus*, comprising more than 800 species, have the characteristic of injecting worms and other prey with venom. These conopeptide toxins, highly diverse in structure and action, are highly potent and specific for their molecular targets (ion channels, receptors, and transporters of the prey’s nervous system), and thus are important research tools and source for drug discovery. Next-generation sequencing technologies are speeding up the discovery of novel conopeptides in many of these species, but only limited information is available for *Conus spurius*, which inhabits sandy mud. To search for new precursor conopeptides, we analyzed the transcriptome of the venous ducts of *C. spurius* and identified 55 putative conotoxins. Seven were selected for further study and confirmed by Sanger sequencing to belong to the M-superfamily (Sr3.M01 and Sr3.M02), A-superfamily (Sr1.A01 and Sr1.A02), O-superfamily (Sr15.O01), and Con-ikot-ikot (Sr21.CII01 and Sr22.CII02). Six of these have never been reported. To our knowledge, this report is the first to use high-throughput RNA sequencing for the study of the diversity of *C. spurius* conotoxins.

## 1. Introduction

Gastropods of the genus *Conus* are among the many marine invertebrates that produce important compounds with specific biological activity. More than 800 *Conus* species are recognized, all having a sophisticated system to inject a neurotoxic venom that rapidly paralyzes its prey [1]. These venoms are composed of a complex mixture of mostly disulfide-rich neurotoxic peptides with 10–30 residues, commonly known as conotoxins (or conopeptides), that affect the central and peripheral nervous systems [2]. 

Currently, more than 2000 nucleotide sequences and 8000 peptide sequences of conotoxins have been published, but to date, less than 0.1% have been characterized at the level of their molecular targets [3,4]. Based on the similarity of their signal peptide regions, conotoxins have been categorized into more than 30 gene superfamilies: A, B_1_, B_2_, B_3_, C, D, E, F, G, H, I, I_1_, I_2_, I_3_, J, K, L, M, N, O_1_, O_2_, O_3_, P, Q, R, S, T, U, V, Y, Con-ikot-ikots, ConoCAPs, Conopressins, Conkunitzins, and Conodipins [5,6]. Each gene superfamily can include toxins belonging to different pharmacological families, defined by their molecular targets and pharmacological activities over them [4,5]; however, several distinct gene superfamilies have been shown to contain members belonging to one or more particular pharmacological families. Their structures and functions are highly diverse, and they primarily target membrane proteins, in particular ion channels, membrane receptors and transporters [7,8]. The conotoxin open reading frame (ORF) generally consists of a signal sequence named the pre region, an intervening pro region called sometimes the propeptide, the mature peptide region, and, sometimes, a region located after the mature peptide that is excised out during maturation [5,6,7,8,9]. These peptide toxins have been the subject of considerable attention, including their utility as molecular tools in the field of physiology, largely due to their high potency and specificity on human ion channels [10,11]. These same properties confer them potential for important clinical applications in their native form or as models for drug design. Two examples of the utility and potential for clinical application, respectively, are MVIIA (ziconotide) conopeptide isolated from *Conus magus* to treat chronic pain in patients with severe cancer or AIDS [12,13] and α-conotoxin (Vc1.1) from *Conus victoriae* to treat intense, chronic neuropathic pain [14].

*Conus spurius* is distributed along the coast of the Gulf of Mexico and its diet is based on wandering polychaetes and hemichordates [15]. It has been reported that *C. spurius* produce toxins in several gene superfamilies, such as, for example, I_2_ (κ-conotoxins) [16,17], A- (α-conotoxins) [18], O_1_ [19], and T [20], and other conopeptides not yet classified into superfamilies, such as conorfamides [21,22]. Because next-generation sequencing approaches, such as transcriptomics, have proven useful for rapid discovery of new conopeptide sequences in several *Conus* species [4], here we used RNA-Seq analysis to identify new conopeptides of *C. spurius*.

We identified 80 amino acid (aa) sequences, for which only 55 putative conotoxins were assigned to a known superfamily. Seven of these were selected to validate the bioinformatics analyses through RT-PCR sequencing. This omics approach enabled the discovery of six novel conotoxin sequences with biotechnological potential.

## 2. Results

### 2.1. Putative Conopeptide Precursors Predicted by ConoSorter

Around 156,215,000 raw reads were assembled using Trinity software, yielding 141,629 transcripts with a mean length of 588.31 base pairs (bp), which were analyzed with ConoSorter. In the Regular Expression file generated by ConoSorter, 52,457 putative conopeptide precursor protein sequences were identified from all possible translations of the assembled sequences using six reading frames, and 3,642 transcripts of conopeptides in the pHMM file. A total of 56,099 amino acid sequences obtained from ConoSorter were filtered according to Prashanth and Lewis [23] criteria, resulting in a total of 4310 putative conopeptide precursors. Subsequently, in a BlastX search using Blast2GO software, 318 amino acid sequences (7.3%) were annotated, with only 80 peptide sequences having average sequence identity >50% with conotoxins related to a species of *Conus*.

The 80 peptides were then classified using Blast2GO, and the cleavage sites were predicted using the ConoPrec tools at the ConoServer website, and 55 of these corresponded to putative conopeptides (3, A-Superfamily; 8, Con-Ikot-Ikot-Superfamily; 3, ConoInsulin-Superfamily; 3, Conophysin-conopressin; 1, Conotoxin-Specific Protein DiSulfide Isomerase (CSPDI); 4, I-Superfamily; 4, L-Superfamily; 4, M-Superfamily; 8, O-Superfamily; 1, P-Superfamily; 2, Q-Superfamily; 1, S-Superfamily; 11, T-Superfamily; 1, W-Superfamily and 1, Z-Superfamily), three correspond to other types of peptides (2, beta-defensin 50 and 1 neuropeptide FF receptor 2-like), and 22 amino acid sequences of unknown classification (Table S1).

### 2.2. Confirmation by RT-PCR and Classification of Conotoxins

Of the 55 conotoxins classified, seven amino acid sequences (aa) (DN55915c1g1i844, DN55915c1g1i644, DN55576c0g3i132, DN55798c5g2i322, DN25679c0g1i142, DN53806c4g4i124, and DN55158c1g2i934) were randomly selected and amplified by RT-PCR, and then sequenced by the Sanger method (Table 1). The comparison of both types of nucleotide sequences (Sanger sequencing vs. assembled reads) showed > 92% similarity for six conotoxin ORF genes and one with 49% similarity (DN55158c1g2i934).

The conopeptide sequences were manually reanalyzed using a Blast sequence identity search and ConoPrec tools, where three sequences were corrected (DN55576c0g3i132, A-Superfamily; DN53806c4g4i124, Con-ikot-ikot conopeptide, and DN55158c1g2i934, Con-ikot-ikot conopeptide).

**Figure 1:**
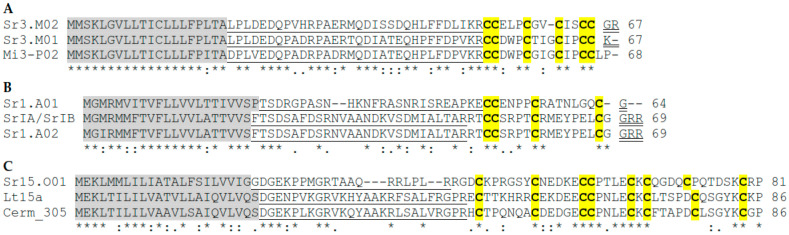
Alignment of the new conopeptide precursors identified from the venom duct transcriptome of *C. spurius.* Alignment of the M-superfamily Sr3.M01 and Sr3.M02 precursors from *C. spurius* with Mi3-P02 of *C. miles* (**A**), alignment of A-superfamily Sr1.A01 and Sr1.A01precursors from *C. spurius* with SrIA/SrIB previously reported for *C. spurius* (**B**), and alignment of O_2_-superfamily precursors Sr15.O01 from *C. spurius* with O_2_-superfamily precursors Cerm_305 of *C. ermineus* and Lt15a precursors of *Conus literatus* (**C**). The signal regions are highlighted in gray; propeptides are underlined; Cys residues of mature toxins are in bold and yellow-shaded; ‘‘post’’ peptides are double-underlined.

Here, we focus on describing only the validated conotoxins and each identified superfamily. M-superfamily conotoxins have eight cysteine (Cys) frameworks (I, II, III, IV, VI/VII, IX, XIV, and XVI) and include three distinct pharmacological families, μ-conotoxins, κM-conotoxins, and ψ-conotoxins, blocking voltage-gated sodium channels, voltage-gated potassium channels, and nicotinic acetylcholine receptors, respectively [3,4,5]. In this study, two M-superfamily precursors (Sr3.M01 and Sr3.M02) presented the Cys framework III and have similarity with Mi3-P02 precursors of *Conus miles* [24] (Figure 1A).

Until now, A-superfamily conotoxins have been shown to contain six Cys frameworks (I, II, IV, VI/VII, XIV, and XXII) and to affect at least one of these three targets: nicotinic acetylcholine receptors (nAChRs) subtypes, the GABA_B_ receptor, and the α1-adrenoceptor [3,4,5]. We identified two conopeptides (Sr1.A01 and Sr1.A02) with cysteine framework I (CC-C-C). The Sr1.A02 conopeptide is similar to the α-conotoxins SrIA and SrIB previously reported by López-Vera et al. [18]. The only difference in Sr1.A02 is the Ile residue in the third position of the signal peptide. However, this synonymous variant does not affect changes in the mature toxins (Figure 1B).

The O-superfamily conotoxins are composed of four Cys frameworks (XII, XV, VI, and VII) and classified as δ, µO, ω, κ, and γ families [3,4]. One conopeptide O_2_-superfamily (Sr15.O01) was identified as sharing cysteine framework XV. The mature protein contains an arrangement of eight Cys residues. However, the arrangement of eight Cys residues differs from other the O_2_ superfamily from other *Conus* species as Cerm_305 precursors from *Conus ermineus* [25] and Lt15a precursors of *C. litteratus* both with eight Cys residues in the mature toxin but at different positions [26] (Figure 1C).

**Figure 2:**
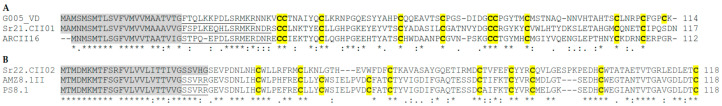
Alignment of one of the Con-ikot-ikot precursors identified (Sr21. CII01) from *C. spurius* with Con-ikot-ikot precursors (G005_VD) of *C. geographus* and Con-ikot-ikot precursors (ARCII16) of *C. arenatus* (**A**), alignment of precursor identified (Sr22.CII02) from *C. spurius* with Con-ikot-ikot precursors AMZ8.1II from *C. andremenezi* and PS8.1 from *C. praecellens* (**B**). Signal regions are highlighted in gray. The ‘‘pro’’ peptides are underlined, the Cys residues of mature toxins are in bold and yellow-shaded.

Con-ikot-ikot toxin (CII) was identified for the first time in *Conus striatus*, one of the most common species of piscivorous cone snails, and has an effect on AMPA receptors, inhibiting channel desensitization [27]. In *C. spurius*, we identified two Con-ikot-ikot precursors (Sr21.CII01 and Sr22.CII02): the Sr21.CII01 conopeptide precursor contains a mature toxin with 77 amino acid residues (aa) and 10 Cys residues. Alignment showed 50% identity with the cysteine frameworks of the G005_VD precursor from *Conus geographus* [28] and ARCII16 precursor from *Conus arenatus* [29] (Figure 2A).

The conopeptide precursor Sr22.CII02 yields a mature toxin with 90 aa residues and eight Cys residues. The Blast search showed that precursor Sr22.CII02 shares 52% similarity with the Con-ikot-ikot precursors from two sister species, AMZ8.1II from *Conus andremenezi* and PS8.1 from *Conus praecellens*, with 10-Cys residues frameworks [30]. The alignment of these three sequences shows that they share the MTMDMKMTFS sequence in the signal peptide; however, the precursor from *C. spurius* lacks a propeptide region (Figure 2B).

## 3. Discussion

The ConoSorter algorithm has been used to identify conotoxin precursors from RNA-seq analysis of transcriptome of several *Conus* species, after assembly by Trinity and other algorithms. For example, in *C. marmoreus* [31], 158 novel conopeptide precursors were identified, and 106 of these were validated by protein mass spectrometry and classified among 13 novel gene superfamilies. In an analysis of three venom transcriptome libraries of *C. literatus*, 128 new putative conopeptides were identified and classified into 22 superfamilies [6].

In this work with *C. spurius*, we used the ConoSorter software and subsequently the same characterization pipeline as Prashanth and Lewis [23], where ConoSorter identified 4310 conopeptide precursors. After these sequences were annotated with the Blast2GO software, only 55 putative conopeptides were assigned to a gene superfamily using the ConoPrec tool of the ConoServer website. We also found three peptide sequences (neuropeptide FF receptor 2-like and two beta-defensins 50) that did not meet the characteristics of conotoxins; we also found 22 amino acid sequences that could not be assigned to a known superfamily, so they may belong to novel superfamilies not yet reported. The number of identified putative conopeptides is low relative to other studies where two or more transcriptomes were compared [6,32]. This is probably because only one cDNA library of the venom duct was analyzed here. Generally, in high-throughput sequencing analyses of transcriptomes of the *Conus* species, only the peptide sequences that were assembled and subsequently classified with ConoSorter have been reported. In our work, we used Sanger sequencing for in vitro experimental validation of the samples of seven raw cDNAs to confirm their presence [33]. Complete cDNA sequencing eliminates errors in assembly and then leads to a real classification of the conopeptide precursors. Thus, the results reported here allowed us to identify conotoxin sequences that have not previously been reported.

In this first approach using the transcriptome analysis to explore the toxin diversity in *C. spurius*, we focused on describing the conopeptide precursors that were verified by Sanger sequencing. However, the remaining 70 conopeptide sequences hypothetically correspond to conotoxin precursors.

Two M-superfamily precursors (Sr3.M01 and Sr3.M02) have a mature toxin with the same Cys pattern as that of Mi3-IP02 conopeptide precursor from *C. miles*. The mature toxin belongs to the MMSKL clade [24]. Toxins in the MMSK clade are found in *Conus* species that hunt fish, molluscs, and polychaetes, and have retained the common conotoxins from their ancestral *Conus* species [24]. Two A-superfamily precursors (Sr1.A01 and Sr1.A02) found in *C. spurius*, correspond to the alpha conotoxin group (α4/7) [9], conotoxins that preferentially target nAChRs and inhibit neuromuscular transmission and cause paralysis [34]. Alpha conotoxins have also been reported in other species of worm-hunting *Conus* species of the Eastern Pacific, such as *Conus brunneus*, *Conus nux*, and *Conus princeps*, for example [35].

One conotoxin (Sr15. O01) in *C. spurius* has the framework C-C-CC-C-C-C-C, which has been reported in *C. ermineus* [25] and *C. litteratus* [26]. Recently, a precursor with a new Cys framework (C-C-CCC-C-C-C-CC), O2_cal30, was reported for *Californiconus californicus* [36], another predatory sea cone snail.

Sr21.CII01 Con-ikot-ikot precursors with 10 Cyst-residues in the mature toxin that we identified have also been found in other *Conus* snail species, such as *C. geographus* [28], *C. arenatus* [28], and *C. victoriae* [37]. In our manual Blast search, Sr22.CII02 shared >54 similarity with Con-ikot-ikot precursors of *C. praecellens* and *C. andremenezi* [29], which share MTMDMKMTFS residues in the signal peptide. Possibly, Sr22.CII02 precursor is a novel member of the Con-ikot-ikot conopeptides, which then would not be exclusive to *Conus* fish hunters and that block desensitization of AMPA receptors in dendrites of the mammalian hippocampus [27].

Regarding conopeptides previously identified from this species at the protein and/or nucleic acid level, the results reported in this work (Table S1) confirmed the structure of peptides sr5a (Isolate Sr5.T.05) [20,38], a variant of sr7a which differs from it by one out of 32 residues (Isolate Sr6.O.08) [19], α-SrIA/B (Isolate Sr1.A.02) [18], and κ-SrXIA (Isolate Sr11.I.02) [16,17,39]. However, we did not identify any of the conorfamides, CNF-Sr1 [40], CNF-Sr2 [41], or CNF-Sr3 [21,22]. A tentative explanation is that this was because these peptides were purified from specimens collected off the coasts of the State of Yucatan, whereas the transcriptome was determined for individuals captured off the coasts of the State of Veracruz, and intraspecies variation in the expression of conotoxins is well known [42].

## 4. Materials and Methods

### 4.1. Biological Material

Five specimens were collected off the coast at the port of Veracruz, in the Gulf of Mexico in December 2015. The venom duct was excised from each living snail, immediately added to DNA/RNA Shield™ (Zymo Research, Tustin, CA, USA), incubated overnight at 6 °C, and then stored at −70 °C.

### 4.2. RNA Extraction and Library Preparation and Sequencing

RNA was isolated from a pool of venom ducts from five individuals of *C. spurius*, using Trizol Reagent and the manufacturer’s protocols (Invitrogen, Carlsbad, CA, USA). The RNA was treated with Turbo DNA-free (Ambion, Austin, TX, USA). RNA Integrity values (RIN) were > 7.0 and measured using the Agilent 2100 BioAnalyzer system (Agilent Technologies, Santa Clara, CA, USA) with the RNA 6000 Nanochip. RNA samples were processed using the manufacturer’s protocol for NEBNext Ultra RNA Library Prep Kit for Illumina (New England Biolabs, Ipswich, MA, USA) with the NEBNext Poly (A) mRNA Magnetic Isolation Module and the NEBNext Multiplex Oligos for Illumina. Briefly, 10 μL of library (4 nM) was mixed with 10 μL 0.1 N NaOH for 5 min, then the library was diluted to 20 pM in HT1 buffer. Sequencing was performed using Illumina NextSeq500 with 150-cycle High Throughput 2 × 75 cycles run.

### 4.3. De Novo Transcriptome Sequencing and Putative Conopeptide Precursors Predicted by ConoSorter

The raw data obtained from the RNA-seq were first filtered to remove adapters and low-quality reads using the NGS QC Toolkit v2.3.3 software [43] and program IlluQC.pl for Ilumina data using default parameters. Subsequently, the filtered reads were assembled by the de novo assembly package Trinity v2.12.41 [44]. For classifying the conopeptide superfamilies, query data were sorted initially using ConoSorter [31], which translates raw cDNA sequences into six reading frames and extracts sequences from the first start codon in each read to the first subsequent stop codon. The results generated two files, the Regex.tab file containing 52,457 unambiguously identified amino acid sequences and the pHMM.tab file containing 3642 unclassified amino acid sequences considered to be novel peptides. A total of 56,099 amino acid sequences were filtered using the workflow of Prashanth and Lewis [23], adjusting the parameters to number of reads (n ≥ 1), sequence length (50 to 300 amino acids), number of Cys residues (>4), hydrophobicity of the signal region (>50), class score (≥2) and superfamily score (≥1). To eliminate false amino acid sequences in the pHMM.tab file, we applied an e-value cut-off value (superfamily e-value < 0.001). Sequences that had no assignment to a superfamily were discarded. Only 4310 amino acid sequences met the parameters.

### 4.4. Annotation of Conotoxins

The 4310 amino acid sequences classified into various superfamilies were used as queries to align against sequences in the NCBI non-redundant protein database (Nr), with an Expect (E) value ≥0.001 and a 20-hit maximum, using the Blast algorithm with Blast2GO (in the package OmicsBox ver 1.1.164 (BioBam^®^, Valencia, Spain) [45]. The putative conopeptide sequences were predicted using a local reference database of known conopeptides from the ConoServer databases and then examined manually using the ConoPrec tool [46].

### 4.5. Confirmation by RT-PCR

To validate the integrity of sequences assembled by Trinity v2.12.41, the nucleotide sequences for seven of these putative conotoxins were selected and primers designed for the regions flanking the ORFs (Table 2). Polymerase chain reactions (PCRs) were carried out in 50-μL reaction volumes using standard PCR reagents in a mixture containing 20 ng cDNA (remainder of the library), 1× Reaction Buffer, 2 mM MgCl_2_, 0.3 of each dNTP, 3 μM of each primer, 1 U of Taq DNA polymerase (Invitrogen^TM^, Carlsbad, CA, USA). The thermocycling conditions in the C1000 Touch^TM^ thermocycler (Bio-Rad, Hercules, CA, USA) were 5 min at 95 °C for initial denaturation; 35 cycles of 94 °C for 40 s, 60 °C for 40 s, 72 °C for 45 s; and a final extension of 72 °C for 5 min.

The PCR products were cloned into pCR^®^-TOPO^®^ Vectors (Invitrogen^TM^, Carlsbad, CA, USA) via TA cloning and inserted into electrocompetent *E. coli* cells DH5α (Invitrogen^TM^, Carlsbad, CA, USA). Once clones were randomly selected for cDNA purification and sequencing, plasmids were purified using ZR Plasmid Miniprep (Zymo Research, Irvine, CA, USA) and were sequenced in both senses using the dideoxy chain termination method on a 3730 × l DNA Analyzer (Applied Biosystems, Foster, CA, USA) at the Laboratorio Nacional de Genómica para la Biodiversidad, CINVESTAV-Irapuato (Irapuato, Gto, Mexico).

## 5. Conclusions

Using high-throughput sequencing analysis and a subsequent in vitro validation, we identified 55 new conopeptides from *C. spurius*, distributed in 11 superfamilies (A, I, L, M, O, P, Q, S, T, W, and Z) and four groups (con-ikot-ikot, conoinsulin, conophysin-conopressin, and conotoxin-specific protein disulfide isomerase). We also reported the presence of other peptides, such as beta-defensin 50, which shares 100% similarity with the sequence reported from rat (*Rattus norvegicus*) and the neuropeptide FF receptor 2-like peptide reported from the snail *Biomphalaria glabrata*. Twenty-two of the new conotoxins have not been assigned to a particular superfamily because of a lack of information on their corresponding signal peptide sequences or because they are new superfamilies that are not yet reported in the databases. This study demonstrated the usefulness of applying a transcriptomic approach and molecular assays to discover novel conopeptides in a poorly studied species. This is the first time that these conopeptide sequences have been reported, which contributes to the expansion of the knowledge of *C. spurius* conotoxins.

## Figures and Tables

**Table 1:** Seven putative conopeptide precursors of *C. spurius*, confirmed by RT-PCR.

Precursors	ID	SF	ORF Sequence
Sr3.M01	DN55915 c1g1i844	M	**M**MSKLGVLLTICLLLFPLTALPLDEDQPADRPAERTQDIAT EQHPFFDPVKRCCDWPCTIGCIPCCK
Sr3.M02	DN55915 c1g1i644	M	**M**MSKLGVLLTICLLLFPLTALPLDEDQPVHRPAERMQDISS DQHLFFDLIKRCCELPCGVCISCCGR
Sr1.A01	DN55576 c0g3i132	A	**M**GMRMVITVFLLVVLTTIVVSPTSDRGPASNHKNFRASNR ISREAPKECCENPPCRATNLGQCG
Sr1.A02	DN55798 c5g2i322	A	**M**GIRMMFTVFLLVVLATTVVSFTSDSAFDSRNVAANDK VSDMIALTARRTCCSRPTCRMEYPELCGGRR
Sr15.O01	DN25679 c0g1i142	O	**M**EKLMMLILIATALFSILVVIGGDGEKPPMGRTAAQRRLPL RRGDCKPRGSYCNEDKECCPTLECKCQGDQCPQTDSKCRP
Sr21.CII01	DN53806 c4g4i124	CII *	**M**AMNMSMTLSVFVMVVMAAAVVGFSPLKEQHLSRMKRN DRSCCLNKTYECLLGHPGKEYEYVTSCYADASILCGATNVY DGCCRGYKYCVWLHTYDKSLETAHGMCQNETCIPQSDN
Sr22.CII02	DN55158 c1g2i934	CII *	**M**TMDMKMTFSRFVLVVLITTIVGSSVHGSEVPDNLNHCW LLRFRMCLKNLGTHEVWFDFCTKAVASAYGQETIRMDCT VFEFCYYRCQVLGESPKPEDHCWTATAETVTGRLEDLETC

* Con-Ikot-Ikot conopeptide; in bold letter, start codon Met-residues; SF, conopeptide Superfamily.

**Table 2:** Primer sequences used to amplify putative conopeptide genes using by RT-PCR.

ID-Trinity	Primer Sequence
DN55915c1g1i844	F: 5′-TAAGGCTACTTGCAACAAGGG-3′ R: 5′-AGGACAAGAGGGATCGATAGCAGT-3′
DN55576c0g3i132	F: 5′-ATATAACCATGGGCATGCGGATG-3′ R: 5′-GAAGTCGAGGGCTACTGCAACAT-3′
DN53806c4g4i124	F: 5′-CCCAGAAGGAAACAGAAGAGTTATCG-3′ R: 5′-ACAGGACGTGGCGTGAGGA-3′
DN55798c5g2i322	F: 5′-ATCCAGCTCTGCATTCACCTGAC-3′ R:5′-TCAGAGGGTCCTGGAGTATCAGC-3′
DN55915c1g1i644	F: 5′-CGTGGTCGTGATAACAAAG-3′ R:5′-GAACGCCACAGCTAGGACAAGAG-3′
DN55158c1g2i934	F: 5′-GACACACTGAACAAGGAAGCACA-3′ R:5′-GGTCATGTCAGCACGTTTCCAGA-3′
DN25679c0g1i142	F:5′-CTTTATGTTGGACGGCATG-3′ R:5′-CCGTCGTCTCAGCACAGACATAG-3′

## Data Availability

Not applicable.

## Supplementary Materials

The following are available online at https://www.mdpi.com/article/10.3390/md19100547/s1, Table S1: List of conopeptides precursors identified in *Conus spurius*.

## References

[1] Kohn A.J. (2019). *Conus* envenomation of humans: In fact and fiction. Toxins.

[2] Wu Y., Zheng Y., Tang H. (2016). Identifying the types of ion channel-targeted conotoxins by incorporating new properties of residues into pseudo amino acid composition. BioMed Res. Int..

[3] Gao B., Peng C., Zhu Y., Sun Y., Zhao T., Huang Y., Shi Q. (2018). High throughput identification of novel conotoxins from the vermivorous oak cone snail (*Conus quercinus*) by transcriptome sequencing. Int. J. Mol. Sci..

[4] Fu Y., Li C., Dong S., Wu Y., Zhangsun D., Luo S. (2018). Discovery methodology of novel conotoxins from *Conus* species. Marine. Mar. Drugs.

[5] Robinson S.D., Norton R.S. (2014). Conotoxin gene superfamilies. Mar. Drugs.

[6] Li X., Chen W., Zhangsun D., Luo S. (2020). Diversity of conopeptides and their precursor genes of *Conus litteratus*. Mar. Drugs.

[7] Olivera B.M. (2002). *Conus* venom peptides: Reflections from the biology of clades and species. Annu. Rev. Ecol. Syst..

[8] Jin A.H., Muttenthaler M., Dutertre S., Himaya S.W.A., Kaas Q., Craik D.J., Lewis R.J., Alewood P.F. (2019). Conotoxins: Chemistry and biology. Chem. Rev..

[9] Buczek O., Olivera B.M., Bulaj G. (2004). Propeptide does not act as an intramolecular chaperone but facilitates protein disulfide isomerase-assisted folding of a conotoxin precursor. Biochemistry.

[10] Duggan P.J., Tuck K.L. (2015). Bioactive mimetics of conotoxins and other venom peptides. Toxins.

[11] Duque H.M., Dias S.C., Franco O.L. (2019). Structural and functional analyses of cone snail toxins. Mar. Drugs.

[12] Lee S., Kim Y., Back S.K., Choi H.W., Lee J.Y., Jung H.H., Ryu J.H., Suh H.W., Na H.S., Kim H.J. (2010). Analgesic effect of highly reversible ω-conotoxin FVIA on N type Ca2+ channels. Mol. Pain.

[13] Gao B., Peng C., Yang J., Yi Y., Zhang J., Shi Q. (2017). Cone snails: A big store of conotoxins for novel drug discovery. Toxins.

[14] Pennington M.W., Czerwinski A., Norton R.S. (2018). Peptide therapeutics from venom: Current status and potential. Bioorg. Med. Chem..

[15] Duda J.T.F., Kohn A.J., Palumbi S.R. (2001). Origins of diverse feeding ecologies within *Conus*, a genus of venomous marine gastropods. Biol. J. Linn. Soc..

[16] Aguilar M.B., López-Vera E., Heimer de la Cotera E.P., Falcón A., Olivera B.M., Maillo M. (2007). I-conotoxins in vermivorous species of the West Atlantic: Peptide sr11a from *Conus spurius*. Peptides.

[17] Zamora-Bustillos R., Aguilar M.B., Falcón A. (2010). Identification, by molecular cloning, of a novel type of I_2_-superfamily conotoxin precursor and two novel I_2_-conotoxins from the worm-hunter snail *Conus spurius* from the Gulf of Mexico. Peptides.

[18] López-Vera E., Aguilar M.B., Schiavon E., Marinzi C., Ortiz E., Restano Cassulini R., Batista C.V.F., Possani L.D., Heimer de la Cotera E.P., Peri F. (2007). Novel α-conotoxins from *Conus spurius* and the α-conotoxin EI share high-affinity potentiation and low-affinity inhibition of nicotinic acetylcholine receptors. FEBS J..

[19] Luna-Ramírez K.S., Aguilar M.B., Falcón A., Heimer de la Cotera E.P., Olivera B.M., Maillo M. (2007). An O-conotoxin from the vermivorous *Conus spurius* active on mice and mollusks. Peptides.

[20] Zamora-Bustillos R., Aguilar M.B., Falcón A., Heimer de la Cotera E.P. (2009). Identification, by RT-PCR, of four novel T-1-superfamily conotoxins from the vermivorous snail *Conus spurius* from the Gulf of Mexico. Peptides.

[21] Campos-Lira E., Carrillo E., Aguilar M.B., Gajewiak J., Gómez-Lagunas F., López-Vera E. (2017). Conorfamide-Sr3, a structurally novel specific inhibitor of the Shaker K+ channel. Toxicon.

[22] López-Vera E., Martínez-Hernández L., Aguilar M.B., Carrillo E., Gajewiak J. (2020). Studies of conorfamide-Sr3 on human voltage-gated Kv1 potassium channel subtypes. Mar. Drugs.

[23] Prashanth J.R., Lewis R.J. (2015). An efficient transcriptome analysis pipeline to accelerate venom peptide discovery and characterisation. Toxicon.

[24] Zhou M., Wang L., Wu Y., Zhu X., Feng Y., Chen Z., Li Y., Sun D., Ren Z., Xu A. (2013). Characterizing the evolution and functions of the M-superfamily conotoxins. Toxicon.

[25] Abalde S., Tenorio M.J., Afonso C.M.L., Zardoya R. (2018). Conotoxin diversity in *Chelyconus ermineus* (Born, 1778) and the convergent origin of piscivory in the Atlantic and Indo-Pacific cones. Genome Biol. Evol..

[26] Pi C., Liu J., Peng C., Liu Y., Jiang X., Zhao Y., Tang S., Wang L., Dong M., Chen S. (2006). Diversity and evolution of conotoxins based on gene expression profiling of *Conus litteratus*. Genomics.

[27] Walker C.S., Jensen S., Ellison M., Matta J.A., Lee W.Y., Imperial J.S., Duclos N., Brockie P.J., Madsen D.M., Isaac J.T.R. (2009). A novel *Conus* snail polypeptide causes excitotoxicity by blocking desensitization of AMPA receptors. Curr. Biol..

[28] Dutertre S., Jin A.H., Vetter I., Hamilton B., Sunagar K., Lavergne V., Dutertre V., Fry B.G., Antunes A., Venter D.J. (2014). Evolution of separate predation- and defence-evoked venoms in carnivorous cone snails. Nat. Commun..

[29] Sudewi A.A.R., Susilawathi N.M., Mahardika B.K., Mahendra A.N., Pharmawati M., Phuong M.A., Mahardika G.N. (2009). Selecting potential neuronal drug leads from conotoxins of various venomous marine cone snails in Bali, Indonesia. ACS Omega.

[30] Li Q., Barghi N., Lu A., Fedosov A.E., Bandyopadhyay P.K., Lluisma A.O., Concepcion G.P., Yandell M., Olivera B.M., Safavi-Hemami H. (2017). Divergence of the venom exogene repertoire in two sister species of *Turriconus*. Genome. Biol. Evol.

[31] Lavergne V., Dutertre S., Jin A.H., Lewis R.J., Taft R.J., Alewood P.F. (2013). Systematic interrogation of the *Conus marmoreus* venom duct transcriptome with ConoSorter reveals 158 novel conotoxins and 13 new gene superfamilies. BMC Genom..

[32] Dutt M., Dutertre S., Jin A.H., Lavergne V., Alewood P.F., Lewis R.J. (2019). Venomics reveals venom complexity of the piscivorous cone snail, *Conus tulipa*. Mar. Drugs.

[33] Zhang H., Fu Y., Wang L., Liang A., Chen S., Xu A. (2019). Identifying novel conopepetides from the venom ducts of *Conus litteratus* through integrating transcriptomics and proteomics. J. Proteomics.

[34] Azam L., McIntosh J.M. (2009). Alpha-conotoxins as pharmacological probes of nicotinic acetylcholine receptors. Acta Pharmacol. Sin..

[35] Morales-Gonzalez D., Flores-Martinez E., Zamora-Bustillos R., Rivera-Reyes R., Michel-Morfin J.E., Landa-Jaime V., Falcón A., Aguilar M.B. (2015). Diversity of A-conotoxins of three worm-hunting cone snails (*Conus brunneus*, *Conus nux*, and *Conus princeps*) from the Mexican Pacific coast. Peptides.

[36] Bernaldez-Sarabia J., Figueroa-Montiel A., Duenas S., Cervan-tes-Luevano K., Beltran J.A., Ortiz E., Jimenez S., Possani L.D., Paniagua-Solis J.F., Gonzalez-Canudas J. (2019). The Diversi-fied O-Superfamily in *Californiconus californicus* Presents a Conotoxin with Antimycobacterial Activity. Toxins.

[37] Robinson S.D., Safavi-Hemami H., McIntosh L.D., Purcell A.W., Nor-ton R.S., Papenfuss A.T. (2014). Diversity of conotoxin gene superfamilies in the venomous snail, *Conus victoriae*. PLoS ONE.

[38] Aguilar M.B., Lezama-Monfil L., Maillo M., Pedraza-Lara H., López-Vera E., Heimer de la Cotera E.P. (2006). A biologically active hydrophobic T-1-conotoxin from the venom of *Conus spurius*. Peptides.

[39] Aguilar M.B., Pérez-Reyes L.I., López Z., de la Cotera E.P.H., Falcón A., Ayala C., Galván M., Salvador C., Escobar L.I. (2010). Peptide Sr11a from *Conus spurius* is a novel peptide blocker for Kv1 potassium channels. Peptides.

[40] Maillo M., Aguilar M.B., Lopéz-Vera E., Craig A.G., Bulaj G., Olivera B.M., Heimer De La Cotera E.P. (2002). Conorfamide, a *Conus* Venom Peptide Belonging to the RFamide Family of Neuropeptides. Toxicon.

[41] Aguilar M.B., Luna-Ramírez K.S., Echeverría D., Falcón A., Olivera B.M., Heimer de la Cotera E.P., Maillo M. (2008). Conorfamide-Sr2, a gamma-carboxyglutamate-containing FMRFamide-related peptide from the Venom of *Conus spurius* with activity in mice and mollusks. Peptides.

[42] Rivera-Ortiz J.A., Cano H., Marí F. (2011). Intraspecies variability and conopeptide profiling of the injected venom of *Conus ermineus*. Peptides.

[43] Patel R.K., Jain M. (2012). NGS QC Toolkit: A toolkit for quality control of next generation sequencing data. PLoS ONE.

[44] Grabherr M.G., Haas B.J., Yassour M., Levin J.Z., Thompson D.A., Amit I., Adiconis X., Fan L., Raychowdhury R., Zeng Q. (2013). Trinity: Reconstructing a full-length transcriptome without a genome from RNA-Seq data. Nat. Biotechnol..

[45] Conesa A., Götz S. (2008). Blast2GO: A comprehensive suite for functional analysis in plant genomics. Int. J. Plant Genom..

[46] Kaas Q., Yu R., Jin A.H., Dutertre S., Craik D.J. (2012). ConoServer: Updated content, knowledge, and discovery tools in the conopeptide database. Nucleic Acids Res..

